# The Cultivation of Reticence

**Published:** 1919-03-22

**Authors:** 


### THE MATRONS' AND SISTERS' DEPARTMENT.

### THE CULTIVATION OF RETICENCE.

She is a perfect treasure, the reticent nurse. The doctor knows instinctively that he can clear his mind and think aloud concerning his perplexities in her presence because she will not babble to his patient or betray his misgivings. The patient lets out all her domestic anxieties because she feels safe in confessing that she wants to be shielded for a while from relatives. The friends probe and question in vain, and find themselves departing with civil but vague generalities when they wanted piquant details to spread about. Her own friends never know on what case she is engaged. If brought to bay by a direct question about a patient she has one invariable formula, "I do not know." Like "Not at Home," this valuable remark has ceased to have any intention to deceive. It indicates "I do not intend to tell you," and is recognised in nursing circles as its polite equivalent. The reticent nurse is a well into which many remarkable secrets are dropped, but no one ever brings them to the surface for the best of all reasons—no one guesses they are there.

How did the reticent nurse learn this secret of reticence? In her own affairs she is frank enough, and may be heard chattering away with abandon on secular matters, the fashions, Shakespeare, or the musical glasses. How did she attain to this high level of professional solidity? Ask her and she will tell you that she owes it to the influence of matron and sister in the early days of her training.

During her first impressionable months it was ceaselessly, gravely brought to her notice that the compact of secrecy between herself and the patient and the sister of the ward was one never on any account to be broken. No one was to be her confidant. No breath of the ward doings was ever to go beyond its walls. No comment, grave or gay, was ever to be made on what went on. No whisper of what the surgeon said in her hearing was ever to be retailed, even to her closest friend in the same ward. To the sister alone she might speak concerning the things which came under her notice. To all others the reply was to be "I do not know." And thus from the very beginning the sacredness of silence in regard to her daily duties and her patients was impressed on her as one of the vital matters in her vocation. Reticence thus learned and practised early in her career became an ingrained habit.

Is the priceless value of reticence recognised in the training schools? Is it clearly understood that it is a quality which the influences of the nurse's training either bestow or discourage. Temperament counts, of course. There are women naturally indiscreet, and women who instinctively keep faith. But the majority of women in the training school are neither one nor the other. They are merely on the road to become one or the other. Is sufficient attention paid by the chiefs to the duty of cultivating in their pupils reticence about ward work, patients, the doctors with whom they come in contact? The most that can be said is that if the attempt to inculcate reticence be made, it succeeds too often very badly indeed.

The modern training school, with its freer mental atmosphere, its numerous counter-attractions, and healthy tone, succeeds as a rule in keeping nurses from that vulgar talk of their cases which generates the gossipy type. But this is not nearly enough. Nurses need to be definitely trained in the art of retaining what they see and hear, instead of feeling restless until they have retailed it again. They need to be warned not once nor twice but continually against the bad, perhaps essentially feminine, habit of pouring out to the next comer any distressing or grotesque details which come under their notice. They need to be taught the art of repelling like confidences. They need to be made to recognise alike in repeating and in listening the acme of bad form.

The nurse's profession is not the only one in which success depends on reticence for the establishment of confidence. Who would employ a solicitor heard confiding all the details of a client's case to the first man at his club? If reticence can be taught to the articled clerk, if the secrets of the Navy were safe with thousands of young men, the majority merely "temporary," who had to run the gauntlet of inquisitive friends all through the war, cannot this virtue become also a distinguishing mark of the nursing profession?

New possibilities are opening out before nurses. Their part in the community is widening and gaining every day in dignity. But wider responsibilities demand a higher type of nurse, one above all who can inspire confidence by the control of her tongue. The public looks to the training schools to bring this new type of nursing sister into being.

